# Pediatric anterior skull base tumors: Our experience and review of literature

**DOI:** 10.4103/1817-1745.66663

**Published:** 2010

**Authors:** N. K. Venkataramana, Y. N. Anantheswar

**Affiliations:** Advanced Neuroscience Institute, BGS Global Hospital, Kengeri, India; 1 Department of Plastic Surgery, Manipal Hospital, Airport Road, Bangalore, India

**Keywords:** Skull Base lesions in children, Surgical approaches, Neuronavigation, Complications and Outcomes

## Abstract

Surgery for skull base lesions has advanced considerably in the past few years. The improvement in surgical results could be attributed to the availability of refined imaging modalities, modern technological advances and multidisciplinary team approach. In this report, we present our personal experience in the surgical management of 45 children with a variety of skull base lesions treated over 10 years. This article includes a retrospective analysis of the surgical approaches used and their results with a review of the literature.

## Introduction

In the recent years, skull base surgery has evolved as a subspecialty by itself. The first report in the early 1960s by Ketcham *et al*,[1] on the use of a combined intracranial and transfacial approach to the anterior cranial base to manage cancer of the paranasal sinuses was followed by an operative description of the modern technique of temporal bone resection by Lewis and Page[2] Though the mortality was high with these procedures limiting their acceptability during 1960s and 1970s, it has regained momentum in 1980s due to the quantum change in the overall outcomes.

Several factors contributed to this progression such as newer methods of accurate computer-aided imaging of the cranial base, reliable anesthetic techniques, dynamic studies for the evaluation of cerebral blood flow (CBF and prediction of the safety of carotid artery occlusion and reconstruction), and development of reliable vascularized flaps for covering of large defects and overall understanding of microsurgical anatomy. Cranial-base surgery is now an interdisciplinary field involving neurosurgery, head and neck surgery, neurootology, neuroophthalmology, maxillofacial surgery, and plastic and reconstructive surgery.[3–6]

The purpose of this article is to provide a general review of the techniques most commonly used in anterior skull base surgery and to share our 10 years of experience in this area. For a successful surgical outcome, proper understanding of these techniques and their applications, knowledge of the relevant anatomy and the orientation of neurovascular structures is of paramount importance.

## Materials and Results

**Figure 1:**
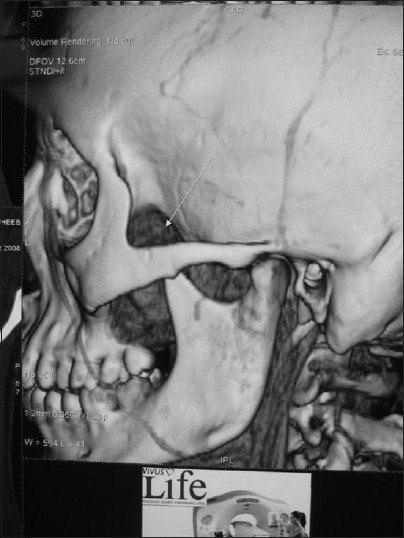
3-D Reconstruction CT scan is useful to assess involvement of bony structures, and for preoperative planning of the skull base tumor surgery

**Figure 2:**
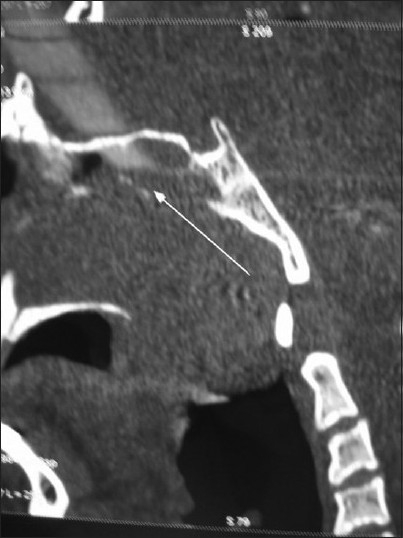
CT scan sagittal reconstruction demonstrating a soft tissue mass (chordoma) extending to the pre clival space and the extent of destruction at the upper part of the clivus and the sphenoid sinus

**Figure 3:**
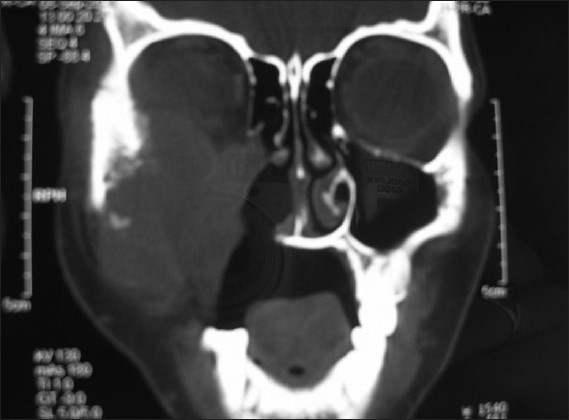
C T coronal view showing a parotid tumor involving the maxillary antrum and extending into the infra temporal fossa, with local bony destruction

**Figure 4:**
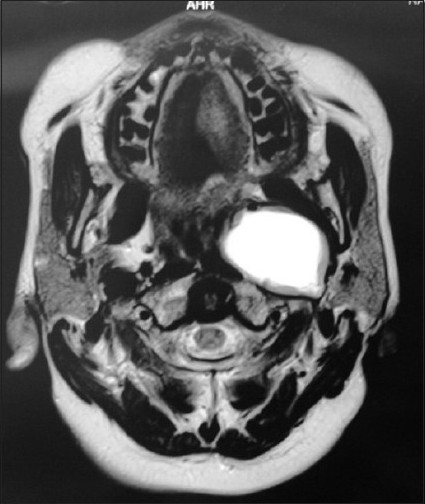
MRI axial view showing a neurofibroma in the left infra temporal fossa

**Figure 5:**
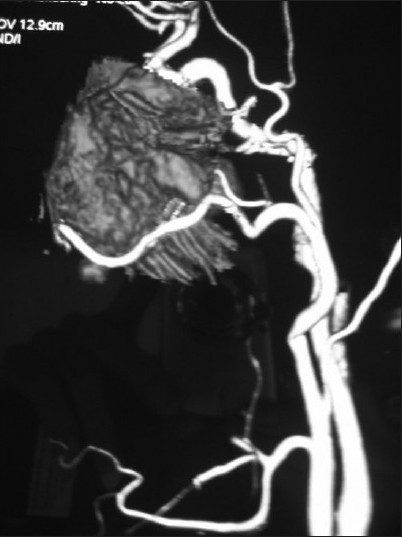
CT angiogram demonstrating normal vascular anatomy with the feeding vessels and vascularity of the tumor

**Figure 6:**
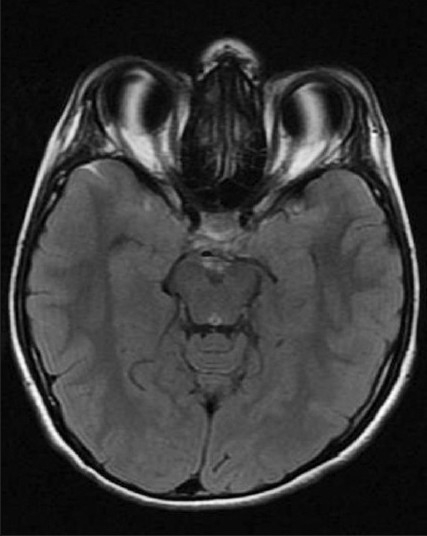
T1-weighted MRI axial view showing a pituitary tumor completely filling the sella in a child

**Figure 7:**
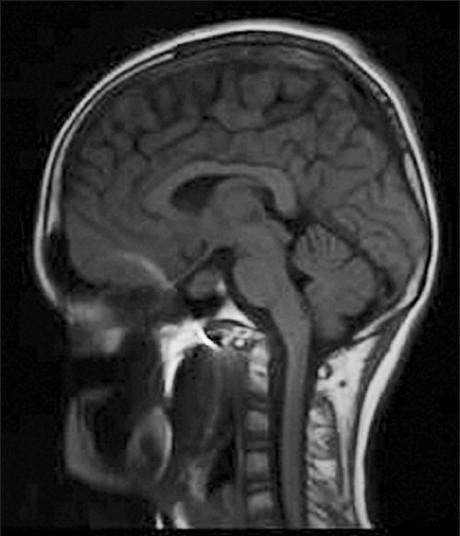
T1-weighted sagittal MRI revealing a pituitary tumor with minimal suprasellar extension in a child

**Figure 8:**
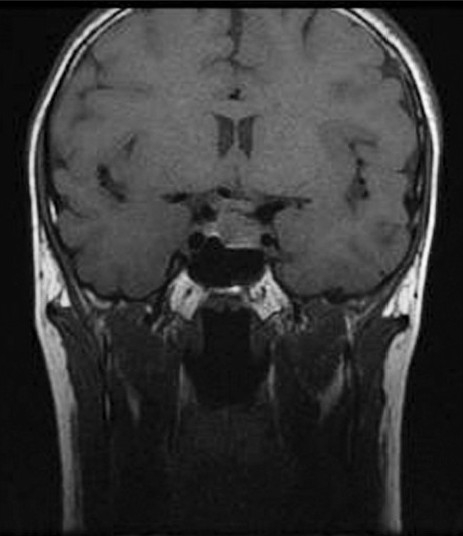
T1-weighted coronal MRI scan showing a pituitary tumor in a child

The hospital record of the children with skull base lesions treated in the departments of neurosurgery and plastic surgery at our institute was analyzed retrospectively. Over a period of 10 years ranging from 2001 to 2010, a total of 48 children with skull base lesions were treated surgically. The age group ranges from 7 to 18 years. The clinical manifestations varied from minor visual symptoms to severe loss of vision, nasal obstruction, mass protruding through the nose, recurrent sinus infection, headaches, and heaviness of the head.[7] Three children had pituitary adenomas and they presented with visual symptoms as well as hormonal disturbances. The location and pathologies are listed in Tables 1 and 2. These children were meticulously examined by the pediatricians, neurosurgeons, plastic surgeons, and anesthetists. Computed tomography (CT) and magnetic resonance imaging (MRI) with 3D reconstructions were performed in all the children [Figures 1–8]. Angiogram was performed in eight children. The parents were counseled about the surgical technique and the possible complications. Different approaches were used based on the size, location, extension, and age of the child. The details of the surgical techniques are described below. Overall, we had very good outcomes. Six children had postoperative complications, with one mortality. The surgical techniques and the complications are described in detail.

**Table 1:** Location of Skullbase Lesions (Our Series)

Location	Number
Fronto-nasal	3
Inter-orbital corridor	13
Pterygomaxillary	15
Infra-temporal	12
Clival	2

**Table 2:** Types of tumors

Pathology	No.
Neurofibroma	1
Clival chordoma	2
Schwannoma	2
Mixed parotid tumor	1
Neuroblastoma	2
Dermoid	2
Pituitary adenoma	3
Fungal granuloma	8
Esthenio-neuroblastoma	12
Angiofibroma	15

Surgical approaches were chosen based on the location and extent of the lesion and age of the child [Table 3].

### 1. Fronto-temporal orbito zygomatic approach: (infra-temporal fossa) [Figures 9–11]

**Table 3:** Cranio-facial approaches

Mandibular swing	2
Lefort-I	8
Modified Weber Ferguson’s	10
Midface splitting	20
Fronto-zygomatico-orbital	5
Transsphenoidal	3
Associated craniotomy	10

**Figure 9:**
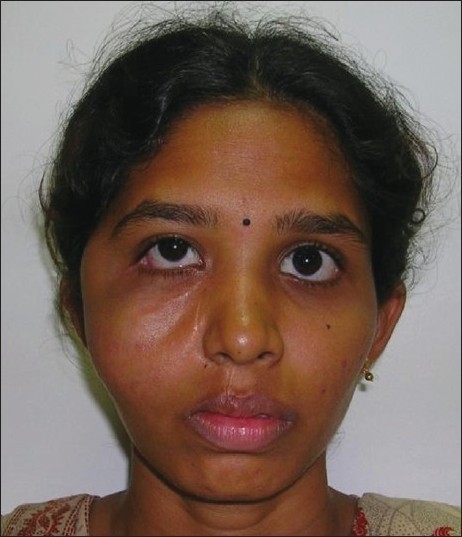
Preoperative clinical photograph of a patient showing a bulge on the right side of the face due to a large parotid tumor with infra temporal extension

**Figure 10:**
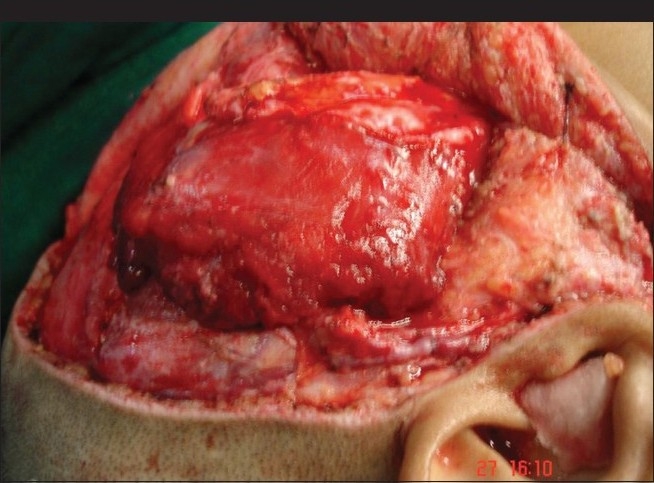
Intraoperative photograph showing extent of surgical exposure of the parotid tumor

**Figure 11:**
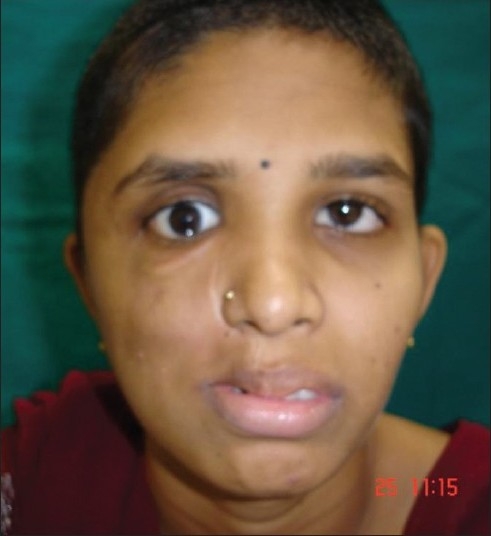
Postoperative clinical picture of the patient after complete tumor removal and reconstruction

A Blair incision is extended superiorly into a hemi-coronal incision. A facial flap is elevated and includes the superficial layer of deep temporal fascia (DTF) which protects the frontal branch of the facial nerve. The zygomatic arch attachment of temporalis is freed allowing access to infra-temporal fossa. Coronoidectomy is performed to prevent postoperative trismus. On completion of the tumor removal, usually the temporalis flap is used to obliterate the defect and the zygomatic arch is repaired with mini plates; however, in this patient with recurrent mixed parotid tumor involving infra-temporal skull base, a microvascular transfer of rectus abdominis muscle was done.[8]

### 2. The midfacial splitting and degloving procedure [Figures 12–16]

**Figure 12:**
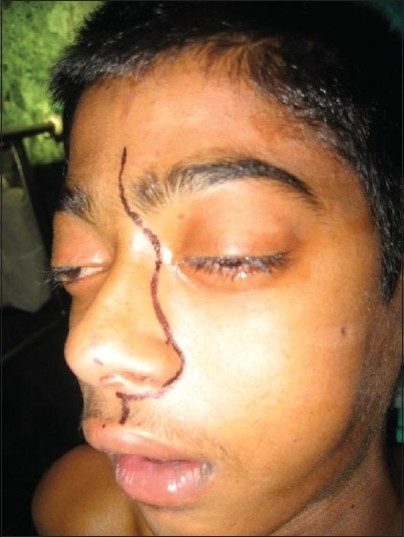
Clinical photograph showing skin incision for the mid face approach used to resect angiofibroma

**Figure 13:**
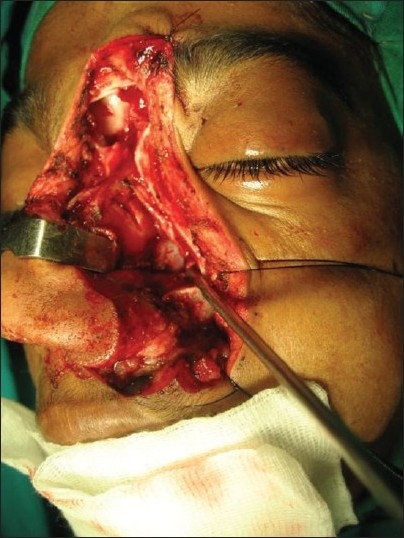
Operative picture showing the extent of the surgical exposure and the cavity after removal of the tumor

**Figure 14:**
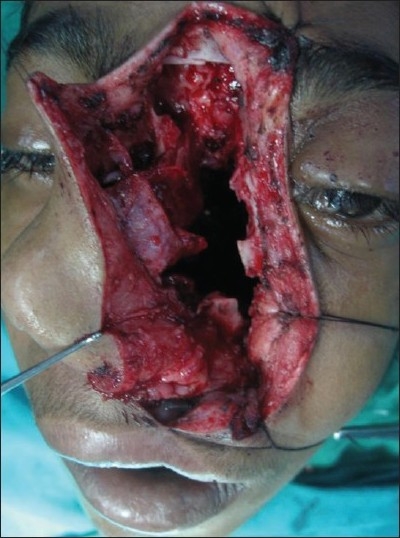
Operative picture showing total removal of the tumor

**Figure 15:**
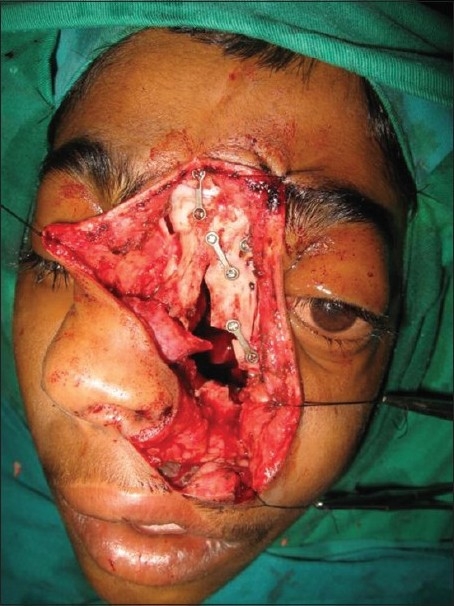
Operative photograph showing reconstruction of mid face using mini plates and screws

**Figure 16:**
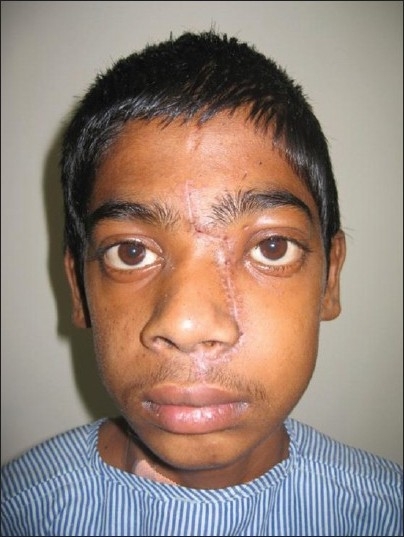
Postoperative clinical picture of the patient at follow-up

The degloving technique provides, when utilized with lateral rhinotomy, wide exposure of the naso-pharynx and medial part of infra-temporal fossa. The incision starts just short of the vermilion border going along the philtral column, alar base, naso-orbital valley, and extending into the frown crease incision into the forehead as required. The skin flap is elevated, which includes the entire soft tissues of the nose bearing the nasal skeleton.

The naso-maxillary skeleton is marked for the required osteotomy lines and then approach osteotomy is made with sagittal saw and completed with fine osteotome. Tumors that invade the frontal sinus, ethmoid roof, superior septum, cribriform plate, floor of anterior and middle fossae, in short, the entire inter-orbital corridor, can be successfully removed by this approach.[9]

After the tumor removal and hemostasis, the osteotomized nasal skeletal segment is replaced and held in position by titanium mini plates and screws. The soft tissues are allowed to return to anatomic position and layered closure is done with 4-0 monocryl and 5-0 prolene.

### 3. Mandibular swing [Figures 17–18]

**Figure 17:**
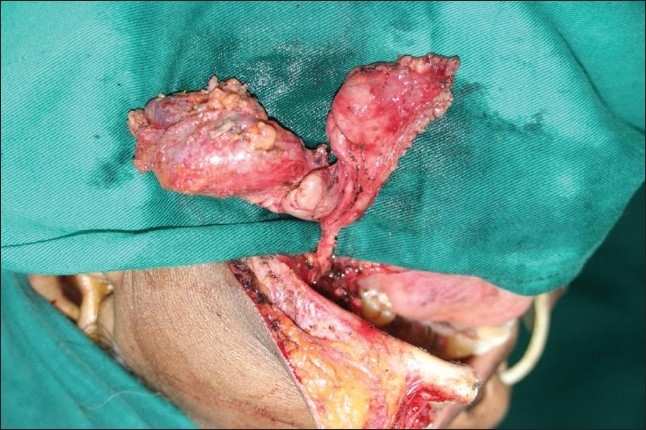
Operative photograph showing the mandubular swing operative exposure to access the skull base

**Figure 18:**
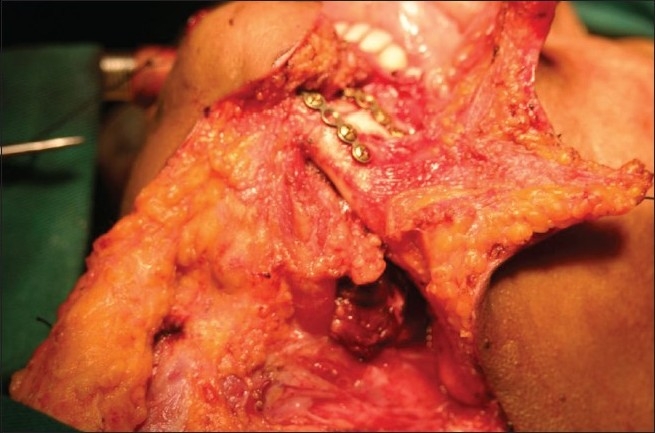
Operative photograph showing the reconstruction of the mandible

This is the earliest approach to reach tumors in the posterior and middle skull base. A lower lip splitting incision carried along the lower border of the mandible going up to the mastoid is used. The cheek flap is elevated laterally. A para mental osteotomy is done after preplating the mandible. The mandible is swung laterally on its hinge at T-M joint allows for the visualization of the tumor in the infra-temporal and sub-mandibular region. In this patient, a bilobed schwannoma of lingual nerve is exposed for resection.

After tumor resection and hemostasis, the osteotomized mandible is placed back in its position and osteosynthesis is carried out with titanium mini plates and screws.

### 4. Le-Forte-I osteotomy approach [Figures 19–22]

**Figure 19:**
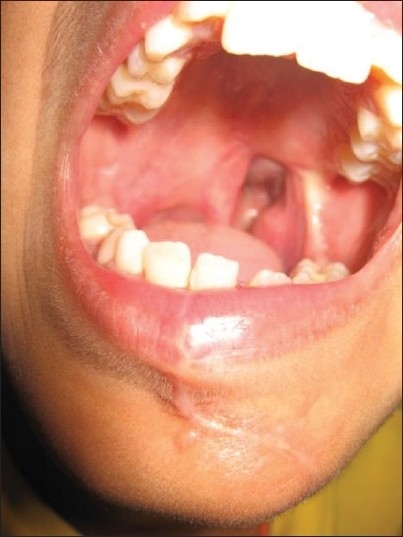
Lefort approach for Chordoma

**Figure 20:**
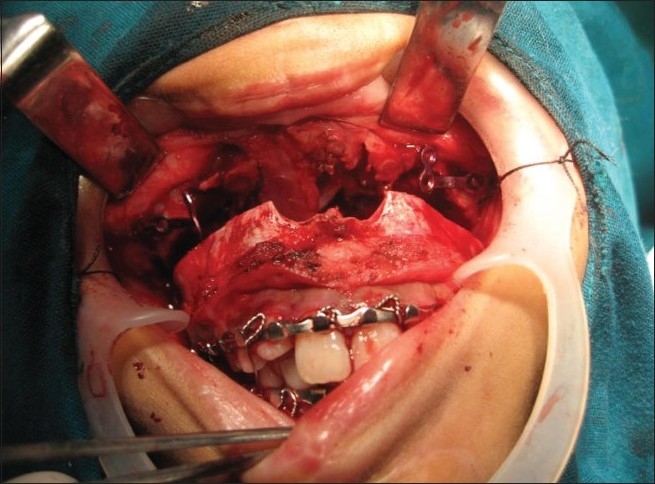
Operative photograph demostrating Lefort approach exposing the supra maxillary structures providing bilateral access to the cranial base

**Figure 21:**
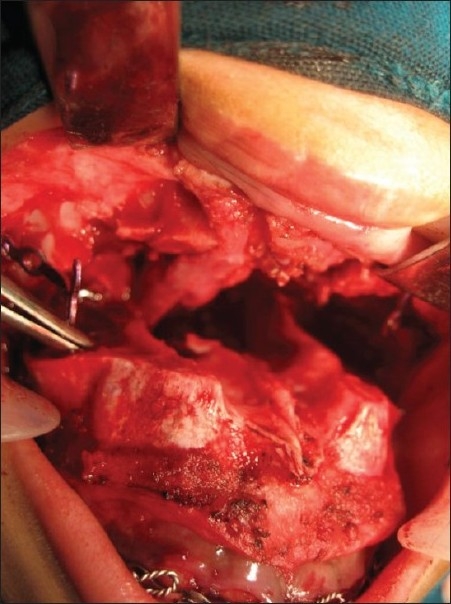
Operative photograph showing removal of the tumor through Lefort approach

**Figure 22:**
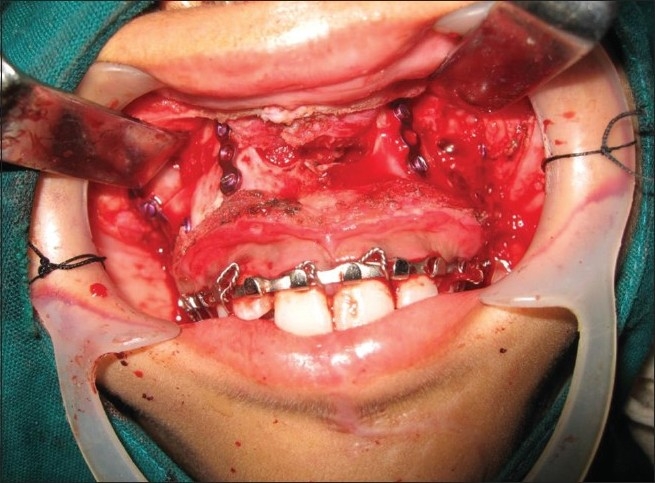
Operative photograph showing reconstruction of facial structures after the Lefort approach

The upper buckle mucosal incision is done from one zygomatico-maxillary buttress to the other. The entire anterior wall of the maxilla around pyriform aperture up to the lateral zygomatico-maxillary buttress is dissected sub-periostally. The osteotomy line is marked at the level of the pyriform aperture. The osteotomy is done from one Z-M buttress to the other Z-M buttress. The nasal spine is osteotomized and the septum is broken up to the posterior extent. The previously applied arch bars provide intermaxillary fixation. After the osteotomy is completed, Howarth’s separator is used to open the broken maxillary segment.[10,11]

This approach provides excellent exposure for tumors that invade the ethmoid roof, superior septum, cribriform plate and sphenoid sinus area. After the tumor extirpation, the hemostasis is achieved and the osteotomized segment is fixed back with ‘L’ shaped titanium mini plates placed along both naso maxillary and zygomatico-maxillary buttresses. Closure of the mucosa is done with 4-0 monocryl.

### 5. Transphenoidal approach

We have used endoscopic transnasal approach for the pituitary tumors. Patient is positioned supine under general anesthesia. The nose is prepared, usually in combination with the ENT surgeons, to get enough corridor by middle talpinectomy and also removal of concha bullosa. Binostril endoscopic technique provides adequate space by mobilization of turbinates, removal of the posterior wall of the septum. The entire Omar and anterior wall sphenoid is removed. Bilateral carotid eminences are identified, the space between them is drilled out to approach the floor of the sella. The mucosa of the sphenoids is removed along with the septae. The dura is exposed from the anterior and posterior intercavernous plexuses. Dura is opened in the midline and enlarged sufficiently to visualize the adenoma. The adenoma is gently removed by mobilizing in all the directions. It is imperative to preserve the normal pituitary gland in children. The stalk and the compressed pituitary is usually seen during the surgery. In large tumors, it may be difficult to identify the normal pituitary gland. Hemostasis is achieved and the repair is done by fascia lata and fibrin glue. A soft nasal pack is kept for 3 days postoperatively. If there is no cerebrospinal fluid (CSF) leak during the surgery, there is no need to keep the external lumbar drain.[12]

## Discussion

Skull base surgery in pediatric population poses some special challenges. These children need proper and meticulous preoperative evaluation and planning. Effective management of blood and fluid volume, electrolyte and temperature management, and good oxegenation are the most critical requirements in both intraoperative and postoperative phases. These children are susceptible to hypoxia, hypothermia, hypoglycemia, and hypo or hypervolemia. Tracheostomy can be performed prophylactically whenever postoperative airway compromise is suspected. Broad spectrum antibiotics and anticonvulsants are routinely administered along with good nutritional supplements. In addition, it is imperative to give attention to many important factors listed below.

### Neuroanatomy

Skull base is a unique area with compact arrangement. Three-dimensional mastery of the relations between the structures is critical to perform safe and successful surgery. Recent understanding of anatomy has paved way through many corridors including endoscopic techniques.

### Imaging studies

CT and MRI are essential in the preoperative planning of cranial-base lesions. These two are often complementary. MRI shows soft tissues in greater detail, but CT is superior in visualizing the craniofacial skeleton and calcified tissues.[13] Multiplanar imaging including reconstructive images and angiography is useful.

Positron emission tomography (PET) is also useful in diagnosis as well as for follow up.

### Vascular evaluation

Cerebrovascular evaluation is essential to determine vascularity and relationship of vascular structures, adequacy of CBF and cross circulation. It also facilitates temporary balloon occlusion (TBO) test and embolization, wherever necessary.

### Intraoperative monitoring

Intraoperative monitoring has proved indispensable in operations on the cranial base,[14] which involves numerous monitoring techniques with varying degrees of usefulness, and neuronavigation to maintain anatomical orientation, cranial nerve monitoring, micro Dopplers contributing to the overall safety [Tables 4 and 5].

**Table 4:** Classification of tumors: Tumors involving the cranial base

Benign	Malignant
*Anterior cranial base*	
Juvenile angiofibroma	Squamous cell carcinoma
Fibroosseous lesions	Esthesioneuroblastoma
*Middle cranial base*	
Meningioma	Nasopharyngeal carcinoma
Pituitary adenoma	Squamous cell carcinoma (paranasal sinus extension)
Chordoma	Adenoid cystic carcinoma
Craniopharyngioma	
*Posterior cranial base*	
Glomus tumors (paraganglioma)	Squamous cell carcinoma (otogenic)
Meningioma	
Chordoma	
Schwannoma, neuroma	

**Table 5:** Foramina of the base of the skull

Foramen	Structures transmitted
Cribriform plate	Olfactory nerve (CN 1)
Foramen cecum	Occasional small vein; origin of sagittal sinus
Optic canal	Optic nerve (CN II); ophthalmic artery
Superior orbital fissure	Cranial nerves III, IV; VI, superior ophthal- mic vein, ophthalmic division of trigeminal nerve
Inferior orbital fissure	Maxillary division of trigeminal nerve filaments from pterygopalatine branch of the maxillary nerve; infra-orbital vessels; anastomosis between inferior ophthalmic vein and pterygoid venous plexus
Foramen rotundum	Maxillary division of trigeminal nerve
Foramen ovale	Mandibular division of tngeminal nerve
Foramen spinosum	Middle meningeal artery
Sulcus tubae auditivae	Lodges cartilaginous part of auditory (eustachian) tube
Foramen lacerum	Closed interiorly by a fibrocartilaginous plate that contains the auditory tube; up- per part traversed by the internal carotid artery
Carotid canal	Internal carotid artery
Stylomastoid foramen	Facial nerve (CN VII); stylomastoid artery
Jugular foramen	Beginning of the internal jugular vein; cranial nerves IX, X, XI
Internal acoustic meatus	Facial nerve (CN VII); vestibulo acoustic nerve (CN VIII)
Hypoglossal canal	Hypoglossal nerve (CN XII)
Foramen magnum	Spinal cord (medulla oblongata); spinal accessory nerves (CN XI); vertebral arter- ies; anterior and posterior spinal arteries; occipito axial ligament

CN: cranial nerve

### Minimally Invasive Techniques of Skull Base Surgery

#### Use of navigation systems

The use of navigation systems has proven to be an invaluable addition both in microsurgery as well as endoscopic surgery of cranial base. Several different systems are now available, allowing the real time feedback to the surgeon. The frameless system is uniquely applicable in pediatric patients. This involves special type of registration software. The CT images are then processed and downloaded onto the computer, where the coronal and sagittal views are reconstructed and displayed simultaneously on a monitor in the operating room along with the endoscopic view. The position of the pre-registered probe or instruments can thus be visualized on the monitor to get real time guidance and orientation. In addition, the navigation system is very helpful in teaching institutions where residents can learn planning and practice proper approaches.[15]

#### Endoscopic approaches

Endonasal cranial-base surgery has now become an established minimally invasive technique for skull base. Improvisation of scopes, microdrills, and instrumentation has opened medial and lateral corridors. This is the procedure of choice especially in midline lesions extending from nasion all the way down to foramen magnum and C2. It takes advantage of the otorhinolaryngologic experience in the functional endoscopic surgery of the nasal and paranasal sinus and the base of the skull with a better view of all median and paramedical anatomic structures. It is less traumatic than the traditional open approaches or the microscopic transsphenoidal approach using the endonasal retractor and postoperative packing. The hospital stay, and hence the surgery cost, are reduced significantly.[16,17]

Although the sinonasal endoscopy was introduced by Hirshmann[18] as early as 1901 by using a modified cystoscope, the first to use the microscope assisted by the endoscope in a transsphenoidal operation was Guiot in 1963.[19] Functional endoscopic sinus surgery was introduced in the 1980s,[20] and endoscopic sinus surgery has been popularized as the surgical treatment of choice for sinonasal problems.[21,22] The use of sinonasal endoscopy has been expanded to include repair of CSF leaks and removal of skull base lesions. The choice of the approach depends mainly on the site and size of the lesion and the surgeon preference in the reconstruction process.[21,23]

The paraseptal approach is made between the middle turbinate and nasal septum; the middle turbinate is displaced laterally, and the nasal septum is detached contralaterally. Bilateral ethmoidectomy is performed to reach the skull base.

The middle meatal approach is made through the middle meatus between the middle turbinate and the orbit. When medial displacement of the middle turbinate, uncinectomy, bilateral ethmoidectomy, and anterior sphenoidectomy are performed, the skull base is exposed. It has a narrower surgical corridor than the paraseptal approach.

The middle turbinectomy approach provides a wider operating corridor leading to the anterior fossa skull base by bilateral ethmoidectomy. Anterior sphenoidectomy provides access to the middle and posterior skull base as well. The loss of supportive structures in the nasal cavity to hold fat grafts and titanium mesh in place can be a drawback of this approach when anterior skull base is reconstructed. The paraseptal and middle meatal approaches are used mainly for lesions on the contralateral side and they are preferred more than the middle turbinectomy approach when reconstruction with a fat graft is expected. Endoscopic endonasal transsphenoidal surgery had been proved safe and effective, in experienced hands, not only for adults but even in children. The minimal invasiveness of this procedure, compared with the transcranial or transsphenoidal microsurgical approaches, makes it ideal for the treatment of several pediatric pathologic conditions such as pituitary adenoma, in which it is essential to preserve the anatomic and functional integrity of hypothalamic pituitary axis, both to assure a normal growth and to maintain good sinonasal viability.[23] This endonasal endoscopy provides excellent surgical exposure from the cribriform plate at the anterior fossa to the foramen magnum, the lower clivus, and upper cervical vertebrae.[24] When an endoscopic endonasal transsphenoidal approach is used in various pituitary lesions, it proves to be advantageous in providing wider exposure at the perisellar region and upper clivus.[^28^]

#### Lesions Involving Cavernous Sinus

Most often, tumors involving the cavernous sinus have been surgically treated through radical surgical approaches such as face-splitting maxillectomy, transcranial lateral skull base approaches, or petrosal approaches. With the use of the endoscopic endonasal transsphenoidal approach, we can manage tumors of anterior skull base and cavernous sinus (chordoma, meningioma, chondrosarcoma, and chondromas) that are not adherent to the carotid artery or dura. The ideal head positioning for the endoscopic endonasal transsphenoidal approach is 15 flexion of the forehead *chin line.[16,17] Endoscopic exposure ranges from the floor of the cribriform plate to the foramen magnum in the vertical dimension and about 15 mm on both sides of the midline between the carotid arteries in the transverse dimension. When an endoscope is adopted during microscopic transsphenoidal surgery, similar visualization may be attainable as well.[24] However, the use of retractors restricts the maneuverability of surgical instruments and restricts the horizontal field dimensions. At the level of the cavernous sinus, the lateral horizontal plane of the field is limited by the carotid artery, but further exposure could be attained up to the foramen ovale and foramen rotundum. At the level of the sellar floor, the abducent nerve lies lateral to the carotid artery, and at the lower clivus, the hypoglossal nerve is the lateral limit. The petrous apex and jugular bulb also are accessible through this endonasal approach. Access to the dorsum sella is somewhat hindered by the pituitary gland. Bone removal was made mainly by the use of high-speed drill. The clival venous plexus can pose problematic bleeding. Dural bleeding is usually controlled by bipolar or monopolar coagulation. Several substances are now available to helphemostasis. The possibility of postoperative CSF leaks and difficulty with reconstruction of the cranial base remain the major potential problems, apart from bleeding in some situations.

#### Reconstruction

Knowledge of the layers of the scalp and its vascular supply is essential in planning surgical incisions that preserve potential reconstructive flaps and maximize primary wound healing. The three most important layers are the pericranium, the galea, and the temporo parietal fascia.[25] The blood supply to the pericranium and galea is primarily from the supraorbital and supratrochlear arteries. The galea also receives its blood supply from the supraorbital and supratrochlear arteries as well as the superficial temporal artery. It can be used alone or in combination with pericranium as a reconstructive flap for the anterior fossa. When a galeal flap is elevated, the risk of causing ischemic necrosis of the forehead skin is present, particularly if the forehead has been exposed to therapeutic radiation. In such situations, cranial-base reconstruction is performed with flaps other than galeal pericranial tissue. If a pericranial flap has been used, a galeal flap cannot be used. The quality, vascularity, and quantity of tissue available from the galea alone for revision reconstruction would be inadequate. It is in this setting that microvascular transfer, usually consisting of a radial forearm or rectus abdominis flap, would be most appropriate. For most patients who have not undergone surgery or radiation therapy, a pericranial flap alone is adequate, and the galea need not be elevated from the overlying cutaneous tissue of the forehead. A bicoronal incision is placed substantially posterior to the hairline so that a sufficient length of pericranium can be preserved anterior to the incision to serve as a useful reconstructive flap.

The other scalp-derived flap for reconstruction of cranial-base defects is the temporoparietal fascial flap. Unlike the temporalis muscle flap, which is a separate entity and is based on the deep temporal vascular system, the temporoparietal fascial flap receives its blood supply from the superficial temporal vessels. Both a temporalis muscle flap and a temporoparietal fascial flap can be raised simultaneously from the same side of the head for reconstruction. The temporoparietal flap is elevated off the subcutaneous tissue of the lateral scalp over the temporal and parietal regions. This flap cannot be used if that area of the scalp has been exposed to therapeutic radiation because of the high probability of scalp loss after removal of the underlying temporoparietal fascial blood supply from the overlying cutaneous tissues. This flap is about 10 cm wide and as long as 16 cm long. It can be tunneled into the nasopharynx, the orbit, the posterior anterior fossa, the middle fossa, and mastoid or temporal defects. The thin, supple nature of the flap coupled with its robust blood supply and minimal donor site morbidity makes it valuable for reconstruction of lateral defects of the cranial base.[25] Unlike transposition of a temporalis muscle flap, use of a temporoparietal fascial flap does not produce contour deformity in the temporal region. Flap survival depends on preservation of the integrity of the superficial temporal vessels during placement of bicoronal incisions and scalp flap elevation [Table 6].

**Table 6:** Approaches to the cranial base

Anterior cranial base
Basilar subfrontal with or without facial incisions (craniofacial approach)
Transfrontal sinus
Middle cranial base
Transoral, transseptal, transsphenoidal approach to the sella
Subtemporal infra-temporal fossa (preauricular)
Subtemporal infra-temporal fossa (postauricular)
Facial translocation
Midfacial split
Facial degloving
Mandibular split procedure
Palatal split procedure
Le Fort I osteotomy with or without transseptal exposure
Posterior cranial base
Extreme lateral approach
Transoral approach to craniovertebral junction
Palatal split procedure
Translabyrinthine approach
Retrosigmoid approach
Suboccipital approach

#### Adjuvant therapies

Radiotherapy is most frequently considered an adjunctive after resection of malignant tumors of the cranial base. Most patients undergoing cranial-base surgery for recurrent malignant disease have received a full course of external beam radiation therapy. In this situation, brachytherapy often is used as an effective method of delivering an additional therapeutic dose to the site of recurrent disease. Brachytherapy relies on implanted percutaneous catheters temporarily loaded with a radioactive source, such as iridium, several days after surgery.[26] Gamma knife or X-knife or stereotactic radiation or radiosurgery can be used after cranial-base tumor resection. The stereotactic beam is guided with CT or MRI data, and it is extremely accurate with respect to the shape and volume of the treated tissue.

Chemotherapy has not proved to be useful in the management of solid tumors of the base of the skull. Pediatric rhabdomyosarcoma, however, has proved chemosensitive. Chemotherapy along with radiation therapy often is used when this tumor occurs at the base of the skull. Biologic modifiers and immunotherapy are under investigation.

### Complications

Variety of complications have been described in the literature [Table 7].[26] Complications in our series are summarized in Table 8.

**Table 7:** Potential complications cranial-base surgery

Category	Complication
Mass lesions	Brain edema
Vascular	Carotid or vertebral artery rupture Arterial thrombosis Arterial dissection Cerebral infarction Venous thrombosis Anticoagulation Induced hypertension Air embolism
Cerebral seizures	
CSF	CSF leakage
Infections	Meningitis Wound abscess Epidural, subdural, or brain abscess
Wound	Flap necrosis local or distant flap repair
Cranial nerve palsy	Cranial nerves I through XII nerve reconstruction
Metabolic	Diabetes insipidus
	Syndrome of inappropriate antidiuretic hormone secretion

**Table 8:** Complications (our series)

CSF fistula	1
Hematoma	2
Ch. osteomyelitis (frontal bone)	1
Diffuse brain edema	1
Death (early post-op)	1

### Cerebrospinal fluid fistula

Postoperative leakage of CSF and the risk of meningitis are the most common complications after cranial-base surgery. As many as 20% of extensive resections of the cranial base entail a CSF leak.[26] To determine the treatment, two primary questions must be answered: weather it is high-flow or low-flow leak and its location.

High-flow CSF leaks usually are managed by means of direct closure as soon as possible. If the location of the leak is not evident, CT cisternography is performed to localize the source. If the leak is low flow, nonoperative measures such as lumbar spinal drain can be tried. If a low-flow leak has not stopped within 5 days, reoperation usually is necessary.

Sometimes it is difficult to determine whether the fluid collected is CSF. This can occur when CSF is leaking into the nasal cavity through the eustachian tubes or through a defect in the floor of the anterior fossa. Fluid that contains glucose probably is CSF because nasal secretions do not contain glucose as does CSF. This test is not specific, however, because serous exudate from the surgical wound often is mixed with nasal mucus in the same area as the suspected leak. The presence of a CSF leak can be most specifically confirmed by means of testing for the protein *≤2-transferrin. Only three body fluids contain this protein: CSF, perilymph, and the vitreous fluid of the eye. The presence of *≤2-transferrin almost confirms the presence of CSF.

### Meningitis

Meningitis is a complication that can follow CSF leak. We always provide prophylactic antibiotics to all patients. The antibiotics will be continued if there is a leak. In the case of meningitis, appropriate antibiotics based on the culture are used. Recurrent meningitis can be a rare complication in undetected CSF leaks.

### Surgical Management of Postoperative Cerebrospinal Fluid Fistula

Unlike spontaneous or posttraumatic CSF fistula, postoperative leaks almost always necessitate surgical closure. Persistent CSF leak after a reasonable wait warrants re-exploration. The technical options for closure depend on factors such as the exact site of the leak, a history of radiation therapy, the size of the defect, the relation of the defect with mucosa-lined cavities, the available reconstructive flaps, and the technical ability of the surgeon.

Although endoscopic closure of CSF fistula is becoming popular, it may not be feasible in acute postoperative leaks. Proper exploration of the resection site and closure or reclosure of areas may be necessary. They often necessitate application of vascularized tissue. The exception is isolated sphenoidal sinus defects usually caused by transsphenoidal hypophysectomy. Artificial dural substances and fibrin glue are of immense help.

In the case of sphenoidal sinus leaks, endoscopic closure with sealing agents such as fibrin glue or hydroxyapatite cement or autografts such as fat and fascia are useful. Fibrin glue is particularly effective in sealing low-flow CSF leaks. High-flow leaks are managed with lumbar drainage before placement of fibrin glue. The fibrin glue immediately seals the leak, and the fat ensures long-term obliteration of the sinus to prevent delayed leakage after resorption of the fibrin glue. Fibrin glue was first used as a hemostatic agent in the early 1900s. It was applied as an adhesive in the 1940s. Since 1980s, highly concentrated forms have been used as a tissue sealant and adhesive. Fibrinolysis can occur when fibrin glue is exposed to excess amounts of plasma. Because the thin layer of scar tissue generated by fibrin glue alone can be eroded by the constant pressure of the CSF pulsations, it is best used with an autogenous fat or fascial graft to enhance long-term stability.

Hydroxyapatite cement can be applied to the small bony defects; it forms a hard barrier to future CSF leakage. Several types of hydroxyapatite cement materials are mixed with a dilute acid aqueous solution that causes setting of the calcium phosphate reactants. Setting usually occurs in 8–15 minutes, depending on the preparation. Once set, these materials stay water sensitive for 4–6 hours and can be dissolved during this period by excess blood or CSF. Hemostasis and temporary (12 hours) use of a lumbar drain is recommended when these materials are applied, regardless of the type of cement. The material must be applied directly to the surface of viable bone after removal of all the surrounding mucosa. This may not be successful for leaks within the ethmoidal sinuses as entrapment of mucosa is unavoidable and success is doubtful.

In addition, repair of dura is equally important. A number of new materials are available such as preserved bovine pericardium and acellular dermis as dural substitutes.

### Postoperative Special Problems

Postoperative cerebral edema, hematoma, vascular emergencies, air embolism, and seizures need immediate attention and treatment. These children require long term follow-up and good rehabilitation especially after reconstructive surgery to enhance their functional capacity.[27]

Vascular complications needs special mention and can cause hypotension, worsening of neurological state. Early recognition can avert the danger. Identification of source is of utmost importance. Direct surgical methods or endovascular methods can be applied to stop the bleeding.

## Conclusion

Skull base surgery is evolving as a specialty through microscopic and endoscopic approaches. Proper imaging, planning, preparation with meticulous surgical technique, and standard intensive care management are mandatory to achieve best results. With the available modern gadgets, majority of skull base lesions can be successfully treated. Team approach and attention to special problems are necessary for pediatric population.
